# The arbuscular mycorrhizal fungus *Rhizophagus clarus* improves physiological tolerance to drought stress in soybean plants

**DOI:** 10.1038/s41598-022-13059-7

**Published:** 2022-05-31

**Authors:** Thales Caetano Oliveira, Juliana Silva Rodrigues Cabral, Leticia Rezende Santana, Germanna Gouveia Tavares, Luan Dionísio Silva Santos, Tiago Prado Paim, Caroline Müller, Fabiano Guimarães Silva, Alan Carlos Costa, Edson Luiz Souchie, Giselle Camargo Mendes

**Affiliations:** 1 Laboratory of Plant Tissue and Culture, Instituto Federal Goiano–Campus Rio Verde, P.O. Box 66, Rio Verde, GO 75901-970 Brazil; 2 Faculty of Agronomy, Universidade de Rio Verde, Fazenda Fontes do Saber-Campus Universitário, P.O Box 104, Rio Verde, GO 75901-970 Brazil; 3 Laboratory of Education in Agriculture Production, Instituto Federal Goiano–Campus Rio Verde, P.O. Box 66, Rio Verde, GO 75901-970 Brazil; 4 Ecophysiology and Plant Productivity Laboratory, Instituto Federal Goiano–Campus Rio Verde, P.O. Box 66, Rio Verde, GO 75901-970 Brazil; 5 Agricultural Microbiology Laboratory, Instituto Federal Goiano–Campus Rio Verde, P.O. Box 66, Rio Verde, GO 75901-970 Brazil; 6 Laboratory of Biotechnology, Instituto Federal de Santa Catarina–Campus Lages, Lages, SC 88506-400 Brazil

**Keywords:** Biotechnology, Plant sciences

## Abstract

Soybean (*Glycine max* L.) is an economically important crop, and is cultivated worldwide, although increasingly long periods of drought have reduced the productivity of this plant. Research has shown that inoculation with arbuscular mycorrhizal fungi (AMF) provides a potential alternative strategy for the mitigation of drought stress. In the present study, we measured the physiological and morphological performance of two soybean cultivars in symbiosis with *Rhizophagus clarus* that were subjected to drought stress (DS). The soybean cultivars Anta82 and Desafio were grown in pots inoculated with *R. clarus*. Drought stress was imposed at the V3 development stage and maintained for 7 days. A control group, with well-irrigated plants and no AMF, was established simultaneously in the greenhouse. The mycorrhizal colonization rate, and the physiological, morphological, and nutritional traits of the plants were recorded at days 3 and 7 after drought stress conditions were implemented. The Anta82 cultivar presented the highest percentage of AMF colonization, and N and K in the leaves, whereas the DS group of the Desafio cultivar had the highest water potential and water use efficiency, and the DS + AMF group had thermal dissipation that permitted higher values of F_v_/F_m_, *A*, and plant height. The results of the principal components analysis demonstrated that both cultivars inoculated with AMF performed similarly under DS to the well-watered plants. These findings indicate that AMF permitted the plant to reduce the impairment of growth and physiological traits caused by drought conditions.

## Introduction

Soybean [*Glycine max* (L.) Merrill] is the world’s most economically important legume^1–4^. However, increasingly frequent periods of water deficit provoked by climate change may cause significant losses in the productivity of the crop^5–7^. Ongoing climate change may result in an increase in global surface temperatures of 1.8–3.6 °C by the end of this century, and constitute one of the greatest challenges faced by human society^8^.

Drought stress interferes directly with the primary metabolism of plants by inducing stomatal closure, which is essential to prevent water loss. This, in turn, limits the input of CO_2_, which inhibits photosynthesis^9–11^, compromising growth and yields^12,13^. Drought also causes an accumulation of reactive oxygen species through an imbalance in the photosystem, which leads to the peroxidation of lipids and the degradation of chlorophyll^14,15^.

A number of different strategies have been tested to mitigate drought stress, particularly the symbiotic association of arbuscular mycorrhizal fungi (AMF) with plants. These fungi are known to exude a glomalin-related soil protein, which contributes to the stability of the soil aggregate and increases the capture of C from the soil^16–19^. Plant-AMF symbiosis also promotes the growth of root hairs through its influence on auxin synthesis and transport^20^. The AMFs are linked to soil properties, which favor the porosity that ensures nutrient uptake^21–23^ and improves the plant-water relationship^24–27^.

The formation of arbuscular structures inside the roots of the host plant provides a pathway for nutritional exchange between the fungus and its host^28^. Hyphae also modulate water transport between the soil and the plant^29,30^. The activation of the lignin synthesis pathway also improves hydraulic transport between cells^15^, increasing transport between the source and sink by expanding the expression of the sucrose transporters^18^.

The accumulation and transport of solutes, in turn, confers osmotic adjustment^31,32^, and antioxidant defense^31,33^, which maintain the plant’s metabolism, even in environments with low water availability. Physiological and metabolic responses have been related to the high productivity of plants when associated with AMF in soil under drought stress^4,27,32,34,35^. Inoculation with AMF may thus provide a potentially valuable strategy for increasing crop productivity and for improving ecosystem sustainability^36,37^. The AMF of the genus *Rhizophagus* (= *Glomus*^38^) can survive and multiply in semi-arid ecosystems, even under conditions of low water availability^39^, and *Rhizophagus clarus* produces larger spores than other species, such as *R. irregularis* and *R. intraradices*^40^. Given this, *R. clarus* has received considerable attention, especially as it can grow symbiotically, using the myristate as a source of carbon and energy^41,42^.

The present study assessed the morphophysiological and nutritional performance of two soybean cultivars, Anta82 and Desafio, which have contrasting levels of tolerance to drought stress, following inoculation and symbiosis with *R. clarus*, which was isolated from soils of the Brazilian Cerrado savanna. The results show that this symbiosis may contribute significantly to the capacity of the plant to tolerate drought stress.

## Results

### Mycorrhizal colonization

**Figure 1:**
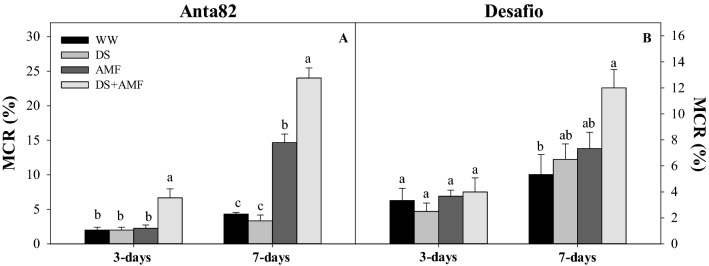
Mycorrhizal colonization (MCR) on days 3 and 7 in the (**A**) Anta82 and (**B**) Desafio soybean cultivars under different conditions of inoculation and water stress. WW = well-watered, noninoculated, DS = drought stress, noninoculated, WW + AMF = well-watered and inoculated, DS + AMF = drought stress and inoculated. The bars represent the mean ± SEM (*n* = 4). Pairs of means in the same period (3 or 7 days) with different letters are significantly different (*p* < 0.05), based on Tukey’s post hoc test.

**Figure 2:**
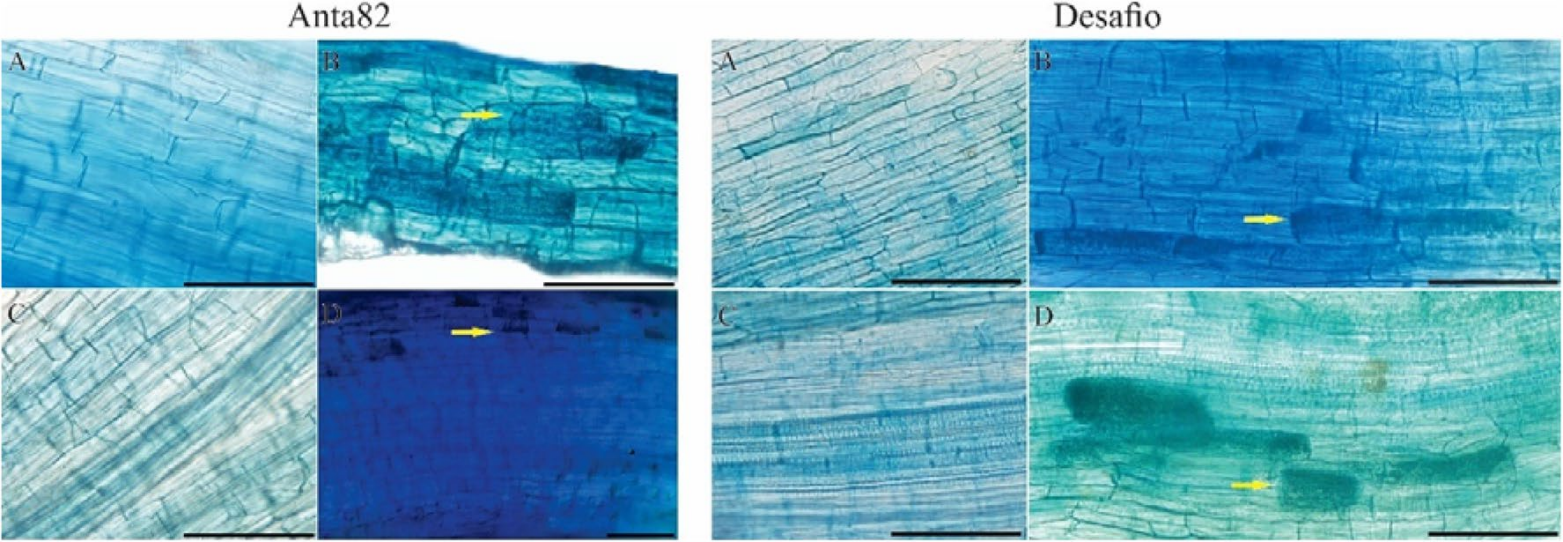
Root cortex in the Anta82 and Desafio soybean cultivars under different conditions of inoculation and water stress. WW = well-watered, noninoculated, DS = drought stress, noninoculated, WW + AMF = well-watered and inoculated, DS + AMF = drought stress and inoculated. The yellow arrows indicate areas of mycorrhizal colonization.

The DS + AMF group of the Anta82 cultivar presented a significantly higher percentage of colonization by *R. clarus* 3 days after the initiation of the drought stress trial (F_3.15_ = 9.22*, p* = 0.0019). On day 7 (F_3.15_ = 84.25*, p* < 0.0001), colonization was significantly higher in both the AMF and the DS + AMF groups (Fig. 1A). In the case of the Desafio cultivar, colonization in the DS + AMF group was significantly higher than that in the WW plants at 7 days (F_3.15_ = 4.64*, p* = 0.0223) (Fig. 1B). The colonization of the soybean root cortex by *R. clarus* arbuscules is shown in Fig. 2.

### Water relations

**Figure 3:**
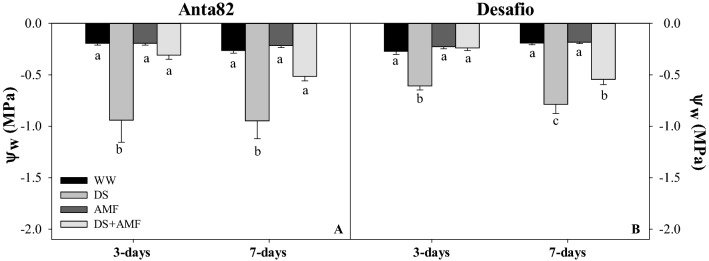
Water potential (Ψ_w_) on days 3 and 7 in the (**A**) Anta82 and (**B**) Desafio soybean cultivars under different conditions of inoculation and water stress. WW = well-watered, noninoculated, DS = drought stress, noninoculated, WW + AMF = well-watered and inoculated, DS + AMF = drought stress and inoculated. The bars represent the mean ± SEM (*n* = 4). Pairs of means in the same period (3 or 7 days) with different letters are significantly different (*p* < 0.05) based on Tukey’s post hoc test.

The plants of the DS group of the Anta82 cultivar presented a water potential (Ψ_w_) of − 0.94 MPa after 3 days of treatment (F_3.15_ = 10.51*, p* = 0.0011), which was significantly lower than all the other treatments (WW, AMF and DS + AMF), which had Ψ_w_ values of no less than − 0.31 MPa (Fig. 3). On day 7, the DS group maintained significantly higher values (Ψ_w_ = − 0.95 MPa), while the DS + AMF plants remained similar to the AMF and WW treatments (Fig. 3).

After 3 days, (F_3.15_ = 38.85*, p* = 0.0001) the DS group of the Desafio cultivar had a significantly lower Ψ_w_ value, than the WW, AMF, and DS + AMF groups, which were, once again all similar to one another (Fig. 3B). At 7 days (F_3.15_ = 32.12*, p* = 0.0001) the DS + AMF had a significantly higher Ψ_w_, of − 0.54 MPa, than the DS group, which had a value of − 0.79 MPa (Fig. 3B).

### Photosynthetic traits

**Figure 4:**
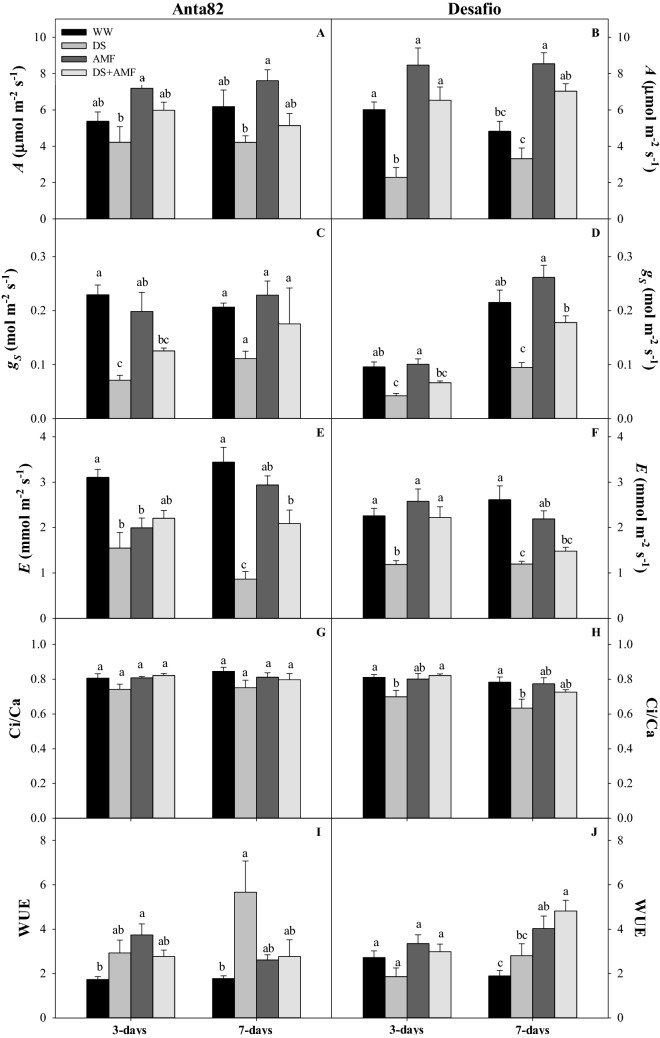
Photosynthetic rate (*A*), stomatal conductance (*g*_S_), transpiration (*E*), the ratio between internal and external CO_2_ concentrations (C_i_/C_a_), and water use efficiency (WUE) on days 3 and 7 in the (**A**,**C**,**E**,**G**,**I**) Anta82 and (**B**,**D**,**F**,**H**,**J**) Desafio soybean cultivars under different conditions of inoculation and water stress. WW = well-watered, noninoculated, DS = drought stress, noninoculated, WW + AMF = well-watered and inoculated, DS + AMF = drought stress and inoculated. The bars represent the mean ± SEM (*n* = 4). Pairs of means in the same period (3 or 7 days) with different letters are significantly different (*p* < 0.05) based on Tukey’s post hoc test.

The photosynthetic rate (*A*) was reduced significantly by drought stress in the Anta82 cultivar on days 3 (F_3.15_ = 4.214*, p* = 0.0298) and 7 (F_3.15_ = 4.80*, p* = 0.0201) (Fig. 4A). The stomatal conductance (*g*_S_) was significantly lower in the DS plants than in the WW plants on day 3 (F_3.15_ = 11.825*, p* = 0.0007) (Fig. 4C). The transpiration rate (*E*) was altered significantly in the DS group on day 7 (F_3.15_ = 18.93*, p* = 0.0001) (Fig. 4E). The C_i_/C_a_ ratio was not affected by either treatment (Fig. 4F). Water use efficiency (WUE) increased significantly in the AMF and DS groups on days 3 (F_3.15_ = 3.988*, p* = 0.0349) and 7 (F_3.15_ = 4.42*, p* = 0.0258), respectively (Fig. 4G). Photosynthetic rates (*A*) varied significantly in the Desafio cultivar on both days 3 (F_3.15_ = 14.206*, p* = 0.0003) and 7 (F_3.15_ = 18.06*, p* = 0.0001) (Fig. 4B). On day 7, the inoculated plants (groups AMF and DS + AMF) presented higher photosynthetic rates in comparison with groups WW and DS (Fig. 4B). The stomatal conductance (*g*_*S*_) was higher in the WW and AMF groups on days 3 (F_3.15_ = 13.59*, p* = 0.0004) and 7 (F_3.15_ = 14.97*, p* = 0.0002) (Fig. 4D), as was the transpiration rate (*E*) on days 3 (F_3.15_ = 8.67*, p* = 0.0025) and 7 (F_3.15_ = 12.427*, p* = 0.0005) (Fig. 4F). The Ci/Ca ratio was lower in the DS plants than in the DS + AMF group on day 3 (Fig. 4H). On day 7, the WUE (F_3.15_ = 7.47*, p* = 0.0044) was significantly higher in the DS + AMF plants than in the WW and DS groups (Fig. 4J).

### Chlorophyll a fluorescence

**Figure 5:**
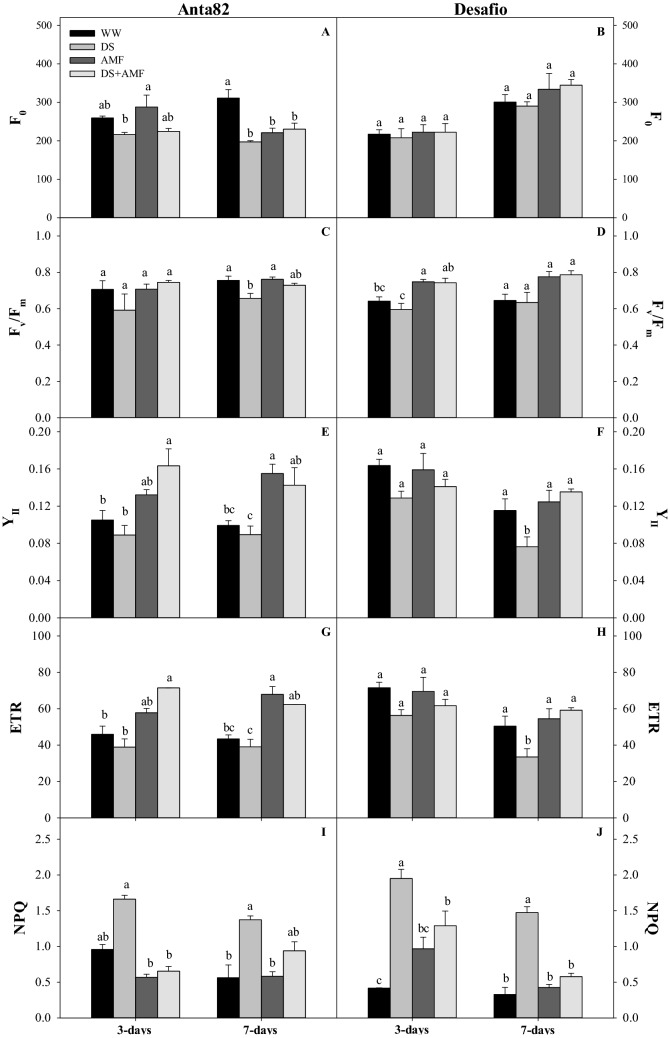
Minimal fluorescence (F_0_), maximum quantum yield of the PSII (F_v_/F_m_), effective quantum yield of PSII (Y_II_), electron transport rate (ETR), and non-photochemical quenching (NPQ) on days 3 and 7 in the (**A**,**C**,**E**,**G**,**I**) Anta82 and (**B**,**D**,**F**,**H**,**J**) Desafio soybean cultivars under different conditions of inoculation and water stress. WW = well-watered, noninoculated, DS = drought stress, noninoculated, WW + AMF = well-watered and inoculated, DS + AMF = drought stress and inoculated. The bars represent the mean ± SEM (*n* = 4). Pairs of means in the same period (3 or 7 days) with different letters are significantly different (*p* < 0.05) based on Tukey’s post hoc test.

The minimum fluorescence (F_0_) was reduced significantly in the DS, AMF, and DS + AMF groups of the Anta82 cultivar after 7 days (F_3.15_ = 11.19; *p* = 0.0008) (Fig. 5A). The maximum quantum yield of PSII (F_v_/F_m_) decreased significantly in the DS group on day 7 (F_Anta3.15_ = 11.195; *p* = 0.0008) (Fig. 5C). Additionally, on day 7, significant reductions were observed in the effective quantum yield of PSII (Y_II_) (F_3.15_ = 7.818; *p* = 0.0037) and the electron transport rate (ETR) (F_3.15_ = 7.341; *p* = 0.0437) of the WW and DS plants in comparison with the AMF and DS + AMF groups (Fig. 5E,G). Non-photochemical quenching (NPQ) increased significantly in the DS plants on both day 3 (F_3.15_ = 71.326*, p* < 0.0001) and day 7 (F_3.15_ = 10.655*, p* = 0.0010) after treatment imposition (Fig. 5I).

In the case of the Desafio cultivar, the minimum fluorescence did not change significantly on either day 3 (F_3.15_ = 0.120; *p* = 0.9469) or day 7 (F_3.15_ = 1.107; *p*  = 0.3845) (Fig. 5B). The maximum quantum yield of PSII increased slightly in the inoculated plants on day 3, although no differences were found among the treatments on day 7 (Fig. 5D). Similarly, neither the effective quantum yield of PSII (F_3.15_ = 2.071, *p* = 0.1577) nor the electron transport rate (F_3.15_ = 2.234, *p* = 0.1368) varied among the treatments after 3 days, although a significant increase was observed in the DS + AMF plants in comparison with the DS group after 7 days (YII: F_3.15_ = 6.077*, p* = 0.009; ETR: F_3.15_ = 6.078*, p* = 0.0093) (Fig. 5F,H). Non-photochemical quenching (NPQ) was significantly higher in the DS and DS + AMF groups than in the WW group on day 3 (F_3.15_ = 9.428; *p* = 0.0018). On day 7, the NPQ of the inoculated plants (AMF and DS + AMF) was no different from that of the WW group (Fig. 5J).

### Photosynthetic pigments

**Figure 6:**
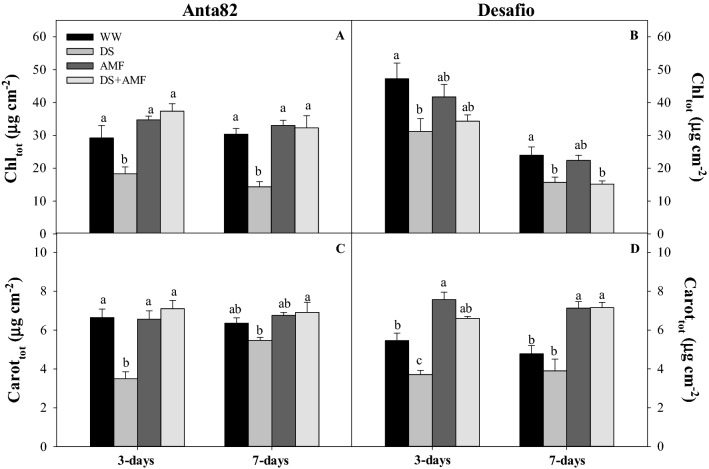
Total chlorophyll (Chl_tot_) and total carotenoid (Carot_tot_) contents on days 3 and 7 in the (**A**,**C**) Anta82 and (**B**,**D**) Desafio soybean cultivars under different conditions of inoculation and water stress. WW = well-watered, noninoculated, DS = drought stress, noninoculated, WW + AMF = well-watered and inoculated, DS + AMF = drought stress and inoculated. The bars represent the mean ± SEM (*n* = 4). Pairs of means in the same period (3 or 7 days) with different letters are significantly different (*p* < 0.05) based on Tukey’s post hoc test.

The total chlorophyll and carotenoid concentrations were reduced significantly in the DS plants of the Anta82 cultivar after 3 (F_3.15_ = 11.16, *p* = 0.0009; F_3.15_ = 15.84, *p* = 0.0002, respectively) and 7 days (F_3.15_ = 14.39, *p* = 0.0003, F_3.15_ = 4.257*, p* = 0.0289, respectively) (Fig. 6A,C). In the cultivar Desafio, total chlorophyll was reduced significantly in the DS and DS + AMF plants in comparison with the WW group on day 7 (F_3.15_ = 15.05*, p* = 0.0002) (Fig. 6B). The carotenoid concentration was significantly higher on days 3 (F_3.15_ = 31.95*, p* = 0.0002) and 7 (F_3.15_ = 15.05*, p* = 0.0002) in the AMF and DW + AMF plants (Fig. 6D).

### Morphological traits

**Figure 7:**
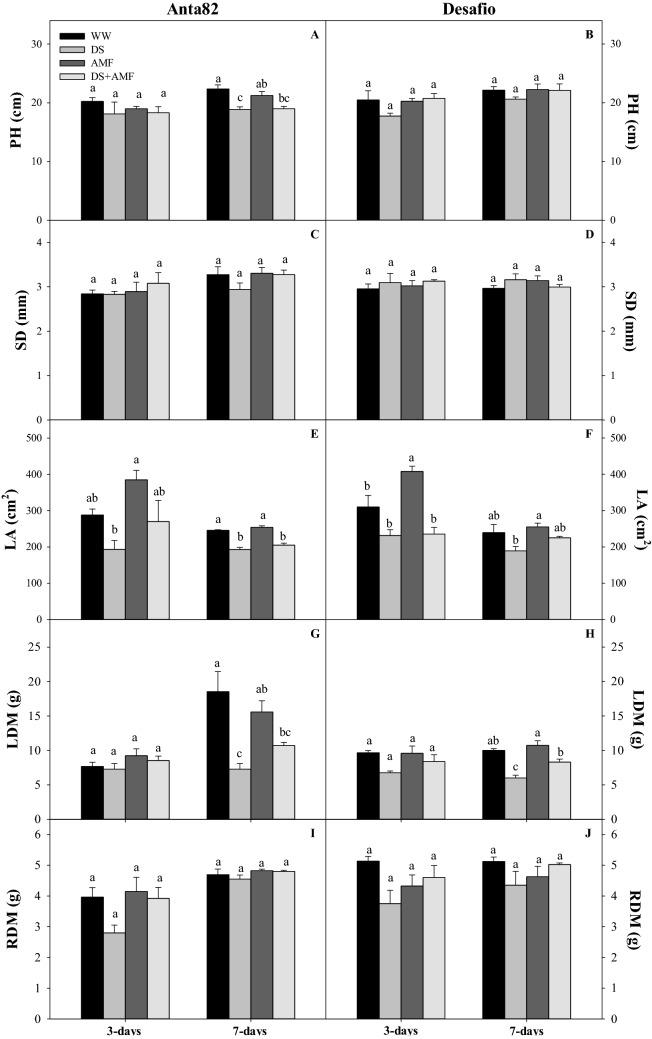
Plant height (PH), stem diameter (SD), leaf area (LA), leaf dry matter (LDM) and root dry matter (RDM) on days 3 and 7 in the (**A**,**C**,**E**,**G**,**I**) Anta82 and (**B**,**D**,**F**,**H**,**J**) Desafio soybean cultivars under different conditions of inoculation and water stress. WW = well-watered, noninoculated, DS = drought stress, noninoculated, WW + AMF = well-watered and inoculated, DS + AMF = drought stress and inoculated. The bars represent the mean ± SEM (*n* = 4). Pairs of means in the same period (3 or 7 days) with different letters are significantly different (*p* < 0.05) based on Tukey’s post hoc test.

In the Anta82 cultivar, plant height (F_3.15_ = 9.421*, p* = 0.0018), leaf area (F_3.15_ = 41.140*, p* < 0.0001), and leaf dry matter (F_3.15_ = 8.385*, p* = 0.0028) were all reduced significantly in the DS and DS + AMF plants in comparison with the WW group on day 7 (Fig. 7A,E,G). Stem diameter and root dry matter were not affected by either treatment in Anta82 on either day 3 (F_3.15_ = 0.475*, p* = 0.7053; F_3.15_ = 3.043*, p* = 0.0704, respectively) or day 7 (F_3.15_ = 1.463*, p* = 0.2740; F_3.15_ = 1.173*, p* = 0.3609) (Fig. 7C,I).

In the case of the Desafio cultivar, by contrast, neither plant height (day 3: F_3.15_ = 2.170*, p* = 0.1446; day 7: F_3.15_ = 0. 945*, p* = 0.4495), stem diameter (day 3: F_3.15_ = 0.336*, p* = 0.7998; day 7: F_3.15_ = 1.124*, p* = 0.3780) and root dry matter (day 3: F_3.15_ = 2.675*, p* = 0.0944; day 7: F_3.15_ = 1.502*, p* = 0.2641) were altered significantly by the treatments (Fig. 7B,D,J). However, leaf area (F_3.15_ = 4.094*, p* = 0.0324) and leaf dry matter (F_3.15_ = 20.058*, p* < 0.0001) increased significantly by day 7 in the inoculated plants (AMF and DS + AMF) in comparison with the WW group (Fig. 7F,H).

### Principal components analyses

**Figure 8:**
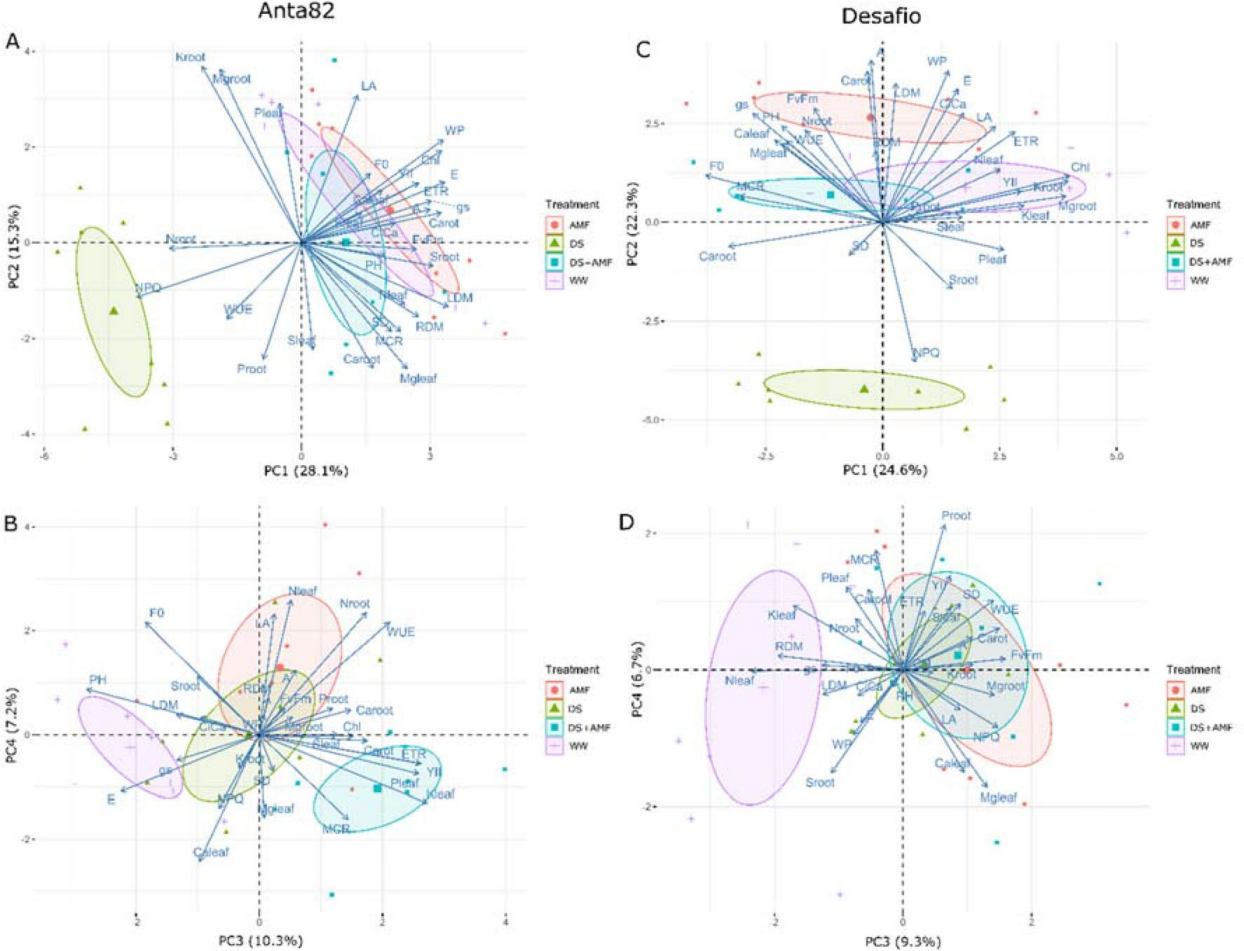
PCA biplot of the first four dimensions (first and second at top and third and fourth at bottom) using (**A**,**B**) Anta82 and (**C**,**D**) Desafio data. and Desafio data at right. WW = well-watered, noninoculated, DS = drought stress, noninoculated, WW + AMF = well-watered and inoculated, DS + AMF = drought stress and inoculated.

The first two components explained 43.4% of the variance in the Anta82 data and 46.9% of that in the Desafio data. The plot of the first two PCA axes clearly differentiated a DS cluster from the other groups in the Anta82 data (Fig. 8A,B).

The first PCA axis of the Desafio data, which explains 24.6% of the variance, is not systematically related to the different treatments (Fig. 8C,D), while the second axis clustered the DS records to the negative side of the plot, the DS + AMF and WW in the middle, and the AMF records toward the positive side. In the case of the Anta82 cultivar, most of the variables (LDM, *E*, Ψw, Chl, Carot, S_root_, *g*_S_, *A*, ETR, RDM, Y_II_, F_v_/F_m_, Mg_leaf_, Nleaf, MCR, C_i_/C_a_, SD, PH, Ca_leaf_, and Ca_root_) were significantly correlated (*p* < 0.05) with the first PCA axis, which means that they decreased with drought stress. In contrast, WUE, Mg_root_, K_root_, N_root_ and NPQ correlated negatively with this axis, which means that these variables increased with drought stress in the Anta82 cultivar.

In the case of the Desafio cultivar, Ψw, Carot, LDM, *E*, F_v_/Fm, Ci/Ca, *g*_S_, PH, LA, Nroot, ETR, Caleaf, WUE, Mgleaf, and RDM were all correlated positively with PC2, while S_root_ and NPQ were correlated negatively with this axis. This means that the former group of variables tends to increase with AMF and decrease with the DS treatments, whereas S_root_ and NPQ increase with DS and decrease with AMF in the Desafio cultivar.

The third axis explained 10.3% of the variance in the Anta82 data and 9.3% of that in the Desafio data. PC3 was thus able to differentiate WW from the other treatments in both cultivars. In the case of the Anta82 cultivar, PC3 also clustered the DS + AMF data. The K_leaf_, Y_II_, ETR, P_leaf_, WUE, Carot, and N_root_ variables were positively correlated with the third PCA axis in the Anta82 data, which means that these variables increase with DS + AMF and decrease with WW, while F_0_, *E* and PH were negatively correlated with this axis.. These variables thus presented the opposite pattern, increasing with WW and decreasing with DS + AMF. In the case of the Desafio cultivar, F_v_/F_m_, Carot, NPQ, M_groot_, WUE, and M_gleaf_ were all positively correlated with PC3, while K_leaf_, RDM, and N_leaf_ were negativelycorrelated . As the WW data were located toward the negative side of the third axis, the former set of variables decreased and the latter increased with this treatment.

Overall, the results of the PCA demonstrated that plants under drought stress colonized with AMF clustered together with the well-watered plants of both cultivars, which clearly indicates that inoculation with AMF contributed to a reduction in the physiological impairment of the plant provoked by drought conditions.

The negative effects of drought also appear to be more pronounced in the Anta82 cultivar than in Desafio. In particular, the third axis of the Anta82 dataset and the second axis of the Desafio data provide insights into the physiological impact of AMF on the plants, independent of their watering regime.

## Discussion

The Anta82 and Desafio soybean cultivars presented differential responses to the inoculation and water treatments. The results of the present study support the hypothesis that inoculation with the arbuscular mycorrhizal fungus *R. clarus* leads to the colonization of the roots of the soybean plant and that, under drought stress, it favors the water status and metabolic activities of the plant, particularly in the case of the more drought-tolerant Desafio cultivar. Arbuscular mycorrhizal fungi are abundant and widely distributed in an ample range of environments and may significantly enhance the stress tolerance of host plants^15^. It is also important to note that the presence of different AMF species is related to the type of host plant and edaphoclimatic conditions^43^. Colonization by mycorrhizae is the principal route of symbiosis with the host plant^44^, which favors both the organisms involved in the relationship. The application of AMF is a potential strategy for the enhancement of the capacity of a plant to tolerate drought in arid ecosystems^45,46^.

The potential for colonization depends on the plant and the type of fungus, in addition to the cultivation conditions and exposure time^47–50^. The Anta82 cultivar is more sensitive to drought stress (DS) and presented an increase in mycorrhizal colonization, especially under drought stress on days 3 and 7. However, the Desafio cultivar presented a pronounced increase in mycorrhizal colonization after 7 days of drought stress. Moreira et al.^51^ found that coffee plants inoculated with *R. clarus* had a higher mycorrhizal colonization (39%) and root dry matter than noninoculated plants, in soils at 71% of field capacity. Inoculation with AMF of different species has also been shown to potentially mitigate the effects of drought stress and increase resistance in bean^52^, rice^53^, tomato^54^, wheat^55^, and in orange^15^ and apple^56^ trees.

The mechanisms associated with the maintenance of the water status of a plant are triggered rapidly when hydrological conditions become limited, which means that drought stress is one of the principal factors affecting plant growth and production^6,57^. These mechanisms include stomatal regulation, which responds rapidly to water stimuli^10,58^. As the root-bound hyphae of mycorrhizal fungi improve water uptake, their association with plants is a potential ally for the maintenance of their water status^47^. Under conditions of drought stress, there is an increase in the space and accumulation of air between the soil particles and the roots, which can be compensated for the presence of AMF, guaranteeing the transport of water^15^. Our results show that AMF mitigated the adverse effects of drought stress in both cultivars. Similarly, the water potential of *Poncirus trifoliata* plants was also increased by 20% under conditions of water deficit when they were inoculated with mycorrhizal fungi^59^. Under drought conditions, AMF helps the host plants to absorb more water, which means that inoculation with these fungi may provide an important strategy for the improvement of the productivity of plants grown in semi-arid regions^60^.

A mechanism that maintains plant turgor, the closure of the stomata may limit photosynthetic processes and compromise crop yields^61–63^. Low photosynthetic yields related to stomatal limitations have been observed in plants exposed to drought stress, although inoculation with AMF increases stomatal conductance under drought stress in comparison with noninoculated plants. Photosynthesis and transpiration were also higher in the presence of mycorrhizae under drought stress conditions in both cultivars in the present study, which indicates that plants associated with AMF can improve their water status under drought stress through their ability to use water more efficiently. Quiroga et al.^64^ found that maize plants in symbiosis with AMF *R. irregularis* under drought conditions had increased stomatal conductance and photosynthesis parameters. Inoculation with AMF may have beneficial effect, even in the absence of drought stress. Coffee plants of three different cultivars, which were well-watered and inoculated with the spores of *R. clarus* and/or *Acaulospora colombiana* presented an increase in photosynthetic rates, stomatal conductance, transpiration, water use efficiency, and the percentage of mycorrhizal colonization^65^. These findings indicate that the AMF had an active role in this process, permitting greater stomatal opening and higher photosynthetic rates, associated with higher turgor, as recently observed in eggplant^27^, tomato^54^, and *Olea europaea*^66^. This was possible because the AMF enhanced water uptake, even under limiting conditions^67,68^, thereby inducing changes in the critical substrate water potential and increasing the water transport in the colonized substrates^26^, which would permit greater water-use efficiency. The maintenance of water status also favors the fixation of atmospheric CO_2_ and increases the movement of photoassimilates (the "sink effect") from the aerial parts of the plant to its roots^48^.

Photosynthetic limitation is directly related to the photochemical responses of the plant, as demonstrated by the chlorophyll *a* fluorescence variables^69^. Drought stress may compromise the functionality of the chloroplast electron chain^9,70,71^, limit the production of energy, and reduce the energy required for the completion of the photosynthetic process. In the present study, the inoculation of soybean plants with AMF under drought stress permitted the maintenance of the maximum quantum yield of PSII (F_v_/F_m_) in comparison with well-watered plants, as observed previously in rice^72^, watermelon^73^, wheat^74^ and maize^14^. By day 3 of the present study, the plants under drought stress used a thermal dissipation mechanism (NPQ) to avoid excess energy expenditure. Thermal dissipation by the xanthophyll cycle, which is activated in response to the pH gradient formed by the cyclic electron cycle^75,76^, is known to be an initial response to abiotic stress and permits the regulation of the amount of excitation energy directed to the reaction centers of the photosystems^77,78^. Higher NPQ rates are important early protection mechanisms for the photosystem, as they avoid the effects of photo-oxidative stress on the photosynthetic photochemical protein complex. The increase in NPQ was efficient in the presence of AMF, especially in plants of the Desafio cultivar, even after 7 days of exposure to drought stress.

The exposure of plants to drought stress can cause an excess of energy expenditure that is not devoted to the photosynthetic process. This results in the increased production of reactive oxygen species (ROS), which promotes the peroxidation of lipids, proteins, and chloroplast pigments^79,80^. There was also a reduction in the concentration of photosynthetic pigments, in particular chlorophyll *a*, which also acts on the photosystem reaction centers. In the present study, drought stress contributed to a reduction in the photosynthetic pigments of the soybean plants, although this damage was mitigated in the plants inoculated with AMF. These results are consistent with the findings of Baslam and Goicoechea^81^, who observed that the association of the AMF *Rhizophagus intraradices* (*Glomus intraradices*) and *Funneliformis mosseae* associated with *Lactuca sativa* plants resulted in an increase in the chlorophyll and carotenoid content, even after exposure to drought stress. In addition, *R. clarus* and a mixture of AMF spores (including those of *R. clarus*) induced an increase in the activity of antioxidant enzymes and the concentration of malondialdehyde in strawberry^82^ and soybean plants^83^ under drought conditions, which may also contribute to the avoidance of oxidative stress and the degradation of pigments.

The impairment of the photochemical stage, associated with the oxidative stress caused by drought stress, may limit plant growth. Under unfavorable water conditions, the plant tends to initially expands its root system to increase the area of contact with the soil^12,84,85^. This compromises aerial growth, including the development of new leaves and shoots^86^. In the present study, even after a short period of stress, it was possible to observe a reduction in the vegetative growth of the soybean plants exposed to drought stress, which was mediated in part in the plants inoculated with *R. clarus*. Inoculation with *Funneliformis geosporus* and *Funneliformis mosseae* contributed to the growth of *Fragaria ananassa* plants under drought stress, as observed by Boyer et al.^87^. Oliveira et al.^88^ found an increase in the yield of *Cicer arietinum* following inoculation with the AMF *Rhizophagus irregularis* and the bacterium *Mesorhizobium mediterraneum* in the absence of drought conditions. Under drought stress, however, these authors observed an increase in plant biomass and the crude protein content of the grains in comparison with the control plants. Bernardo et al.^89^ found that wheat plants (*Triticum* spp.) inoculated with *Funneliformis mosseae* (*Glomus mosseae*) accumulated more dry matter in the aerial parts under drought stress than the noninoculated plants. Plant growth occurs through the accumulation of nutrients, which is enhanced by the interaction between the soybean plants and the AMF. Under drought stress, nutrients may be accumulated in or adsorbed by the roots, due to the reduction in the flow of mass that is normally promoted by transpiration. When associated with AMF, plants increase the efficiency of their water use, which permits the transfer of nutrients to the shoots. The symbiosis between AMF and legumes may be relevant to the rhizobia nodulation of N_2_-fixing bacteria. This interaction may be responsible for nutrient recycling and favor nutrient uptake^67^, as well as improving the tolerance of abiotic stress^90^. *Rhizophagus clarus* is also known to increase the effectiveness of chemical fertilizers in soybean plants under field conditions, and to increase the P and N contents in inoculated plants^91^. This is an economically important finding, given that it would favor a reduction in the application of fertilizers to farm crops.

Overall, inoculation with *R. clarus* increased the capacity of soybean plants to tolerate drought stress, by modifying their metabolism to permit the maintenance of or even an increase in their development under moderate drought conditions. The potential mitigation of the effects of drought stress by *R. clarus* provides important insights for the development of further research, including field experiments to verify the response of the plants under natural conditions, considering the potential occurrence of multiple abiotic stressors, to support the development of more efficient agricultural practices in regions subject to moderate water stress.

## Conclusions

The arbuscular mycorrhizal fungus *Rhizophagus clarus* supported the maintenance of the water status of Anta82, a drought-sensitive soybean cultivar, mitigating the negative effects of drought stress on the photosynthetic apparatus of this plant. In the case of the Desafio cultivar, which is moderately drought-tolerant, greater colonization by *Rhizophagus clarus* increased the concentration of photosynthetic pigments and improved the physiological performance of the plant and its growth. These data indicate that inoculation with *Rhizophagus clarus* is a potentially important tool for the improvement of soybean yields, especially in regions with low precipitation that are subject to drought.

## Materials and methods

### Plant material and experimental conditions

The experiment was conducted in a climated-controlled greenhouse (~ 27 °C and relative humidity of ~ 75%) at the Laboratory of Ecophysiology and Plant Productivity at the Rio Verde campus of the Goiano Federal Institute of Science and Technology in Rio Verde, Goiás, Brazil. In the greenhouse, soybean plants (*Glycine max* (L.) Merrill) of two cultivars—Anta82 RR (Anta82; Geneze Seeds, São Paulo, Brazil), which is drought-sensitive, and the moderately drought-tolerant Desafio 8473 RSF (Desafio; Brasmax Sementes, Cambé, PR, Brazil)—were grown in 3-dm^3^ pots. Each pot contained a mixture (2:1) of Red Latosol (LVdf), which is the typical soil of the Brazilian Cerrado savanna, and sand, which had been corrected to 60% of base saturation with PNRT 100 dolomitic limestone. This substrate was fertilized based on the results of the chemical analysis (Supplementary Table S1) and the recommendation for Cerrado soils^92^.

The arbuscular mycorrhizal fungus (AMF) *Rhizophagus clarus* was donated by the germplasm bank of the Soil Microbiology Laboratory at the Ilha Solteira campus of São Paulo State University (UNESP) in Ilha Solteira, São Paulo, Brazil. To multiply the inoculum, the sand/soil (2:1) substrate was first placed in cotton bags, which were sterilized in an autoclave at 1.5 atm at a temperature of 121 °C for two hours. The substrate was then dried in an oven at 105 °C for 24 h, and rehydrated with distilled and sterilized water for 24 h^93,94^. Each plastic pot (1 dm^3^) containing sterilized substrate received approximately 10 g of the AMF inoculum together with *Sorghum bicolor* seeds, which acted as host plants. After 3 months, the soil was collected and stored in an ultrafreezer prior to use in the experiment. The density of spores was determined by placing a sample of the substrate on an acrylic plate with concentric rings, and examined under a SteREO Discovery.V8 stereomiscroscope (Zeiss, Göttingen, Germany) to count the spores, following Gerdemann and Nicolson^95^ and Jenkins^96^. The spores were stored in an ultrafreezer. For the experiment, each seeding hole in the 3-dm^3^ pots was inoculated with 10 g of the inoculum containing 3.5 g of *R. clarus* spores during the sowing of the soybean^97^.

The experimental was based on a random-block design, with four replicates. Each pot containing four plants was considered to be an experimental unit. The experimental water treatment (drought stress) was applied when the plants reached vegetative developmental stage V3, approximately 30 days after germination, when the water-holding capacity (WHC) was maintained at 60%, based on the gravimetric method. The plants in the control treatment (well-watered) were maintained at 100% WHC. The treatments were as follows: well-watered (WW); well-watered and inoculated with *R. clarus* (AMF); drought stress (DS); and DS plants inoculated with *R. clarus* (DS + AMF). The plants in all four groups were evaluated on days 3 and 7 after the initiation of the treatment.

### Mycorrhizal colonization rate

Mycorrhizal colonization rates were determined following Koskey and Gemma^98^. Root samples (~ 0.4 g), were collected and stored in 50% ethanol. For analysis, the samples were initially immersed in 2% KOH, heated in a stove at 90 °C for 120 min, and then washed in distilled water. The samples were then immersed in 1% HCl for 30 min, washed in water, and stained with 0.05% trypan blue in a lactoglycerol solution (1:1:1 lactic acid, glycerol, water) at 90 °C for 10 min^99^. The root fragments were mounted on microscope slides and observed at a magnification of 200 × under an Olympus BX61 microscope (Tokyo, Japan) to determine the percentage of root volume colonized by the fungus^100^.

### Water potential

The predawn leaf water potential (Ψw) was measured using a Scholander 3005–1414 pressure chamber (Soil moisture Equipment Corp., Goleta, CA, USA).

### Physiological traits

Gas exchange was measured in fully expanded leaves to determine the net photosynthetic assimilation rate (*A*, µmol CO_2_ m^−2^ s^−1^), stomatal conductance (*gs*, mol H_2_O m^−2^ s^−1^), transpiration rate (*E*, mmol H_2_O m^−2^ s^−1^), and the ratio between internal and external CO_2_ concentrations (C_i_/C_a_). The measurements were obtained using an Infrared Gas Analyzer (IRGA; LI-6400xt, Licor, Lincoln, NE, USA). The instantaneous water-use efficiency (WUE, in µmol CO_2_ mmol^−1^ H_2_O) was calculated as the ratio between *A* and *E*. All measurements were obtained under a constant photosynthetic photon flux density (PFFD, 1000 µmol photons m^–2^ s ^−1^) and at the ambient atmospheric CO_2_ concentration (Ca, ~ 400 µmol mol^−1^), temperature (~ 25 °C), and relative humidity (~ 50%).

### Chlorophyll a fluorescence

The fluorescence of chlorophyll was evaluated using a 6400–40 LCF fluorometer coupled to the IRGA. The leaves were initially acclimated in the dark for 40 min to obtain the minimum (*F*_0_) and maximum chlorophyll fluorescence (*F*_m_) values and to calculate the maximum quantum yield of photosystem II (PSII) [*F*_v_/*F*_m_ = (*F*_m_ − *F*_0_)/*F*_m_]. The leaf tissue was then exposed to actinic light and a saturating pulse to obtain the steady-state fluorescence (*F*) and the maximum fluorescence in a light-adapted state (*F*_m_′), respectively. This permitted the determination of the effective quantum yield of PSII [Y_II_ = (*F*_m_′ − *F*)/*F*_m_′], and non-photochemical quenching [NPQ = (*F*_m_ − *F*_m_′)/*F*_m_′]. The Y_II_ values were used to calculate the electron transport rate (*ETR* = Y_II_.PFFD.Leaf_ABS_.0.5), where PFFD is the photon flow (µmol m^−2^ s^−1^) in the leaves, Leaf_*ABS*_ is the fraction of incident light that is absorbed by the leaves, and 0.5 is the excitation energy fraction directed to PSII.

The photosynthetic pigments were extracted from the leaf discs (~ 2-cm^2^) immersed in dimethyl sulfoxide solution with calcium carbonate (40 g L^−1^) in a water bath at 65 °C. After 24 h, the solution absorbance was read at 480.0, 649.1, and 665.1 nm using an Evolution 60S UV–VIS spectrophotometer (Thermo Fisher Scientific, Madison, WI, USA). Chlorophyll *a* (Chl*a* = 12.4.*A*_665._1 – 3.62.*A*_649.1_), chlorophyll *b* (Chl*b* = 25.06.*A*_649.1_ – 6.50.*A*_665.1_), and total carotenoids (Carot = [1000*A*_480_ – 1.29 Chl*a* – 53.78Chl*b*]/220) were calculated following Wellburn^101^ and the pigment concentrations were expressed as µg cm^−2^. Total chlorophyll was obtained by summing Chl*a* and Chl*b*.

### Morphological traits and nutrient content

The plants were measured to determine the plant height (PH, cm) and stem diameter (SD, mm). The leaves were separated out to obtain the leaf area (LA, cm^2^). The leaves and roots were dried to a constant weight at 65 °C in a forced-air circulating oven to obtain the leaf dry matter (LDM, g) and root dry matter (RDM, g).

To quantify the nutrient content of the leaves and roots, the content of the dry material (~ 500 mg) was extracted by nitric-perchloric (3:1) digestion and analysed following Embrapa^102^. Nitrogen (N) was measured by the Kjeldahl titration method using a nitrogen distiller (TE-0364, Tecnal, Piracicaba, Brazil). Phosphorus (P) was determined by the molybdenum blue method, and sulfur (S) was determined by the barium chloride turbidity approach, using molecular absorption spectrophotometry (SP1105, Tecnal, Piracicaba, Brazil). Potassium (K) was analyzed using flame photometry (B462, Tecnal, Piracicaba, Brazil) and calcium (Ca) and magnesium (Mg) were determined using atomic absorption spectrophotometry (SavantAA, GBC Scientific Equipment, Braeside, Australia).

### Statistical analysis

The variation in the data were evaluated using analysis of variance (ANOVA) and pairs of means were compared using Tukey’s post hoc test (*p* < 0.05). These analyses were run in SISVAR software (v. 5.6, Lavras, MG, Brazil).

Principal components analyses (PCA) were run on the whole dataset, and for each cultivar separately. These analyses were run in the *FactoMineR*^103^ and *factoextra*^104^ packages in R software^105^. The data were first scaled using the *scale* function and then analyzed using the *PCA* function. The eigenvalues were evaluated to determine the number of axes that should be evaluated. We used the screen plot to visualize the data, based on Cattell's rule, which states that the components that correspond to the eigenvalues to the left of the straight line (eigenvalues lying on the straight line correspond to random variation) should be retained^106^. To better understand the variables represented in each component, we used the *fviz_contrib* and *dimdesc* functions, which determine the contribution of the variable to each component and its correlation with the component, respectively.

### Ethics approval and consent to participate

Manuscripts reporting on studies do not involve any human participants, human data, or the analysis of human tissue are exempt from these considerations.

### Complies with international, national and/or institutional guidelines

The experimental research reported here complies with all the relevant institutional, national, and international guidelines and legislation.

## Supplementary Information

Supplementary Table S1.

## Data Availability

All the data generated or analyzed during the present study are included in this published manuscript. The raw datasets are available from the corresponding author on reasonable request.
