# Supplementary material for: The arbuscular mycorrhizal fungus Rhizophagus clarus improves physiological tolerance to drought stress in soybean plants
Source: Sci Rep. 2022 May 31;12:9044. doi: 10.1038/s41598-022-13059-7 (PMC9156723; doi:10.1038/s41598-022-13059-7)
Supplement: Supplementary file 1 — Supplementary Table S1. [file 41598_2022_13059_MOESM1_ESM.docx]

**Supplementary Material**

**Table 1S.** Soil analysis (0–20 cm) before experiment installation

| Ca | Mg | Ca+Mg | Al | H+Al | K | K | S | P(mel) | pH | M.O. |
| --- | --- | --- | --- | --- | --- | --- | --- | --- | --- | --- |
| ---------------------cmol_c_ dm^-3^----------------- | | | | | | ---------mg dm^-3^---- | | | CaCl_2_ | g dm^-3^ |
| 0.59 | 0.17 | 0.76 | 0.05 | 1.8 | 0.02 | 9 | 0.8 | 0.9 | 5.8 | 6.2 |
| Na | Fe | Mn | Cu | Zn | B | CTC | SB | v% | m% | Argila |
| ----------------------mg dm^-3^------------------- | | | | | | cmol_c_ dm^-3^ | | Sat. Base | Sat. Al | Text % |
| 1.8 | 118 | 16.7 | 0.5 | 0.2 | 0.10 | 2.6 | 0.81 | 30 | 6 | 39 |
